# Supplementary material for: Memantine Disrupts Motor Coordination through Anxiety-like Behavior in CD1 Mice
Source: Brain Sci. 2022 Apr 13;12(4):495. doi: 10.3390/brainsci12040495 (PMC9027563; doi:10.3390/brainsci12040495)
Supplement: Supplementary file 1 [file brainsci-12-00495-s001.zip › Figure S1.docx]

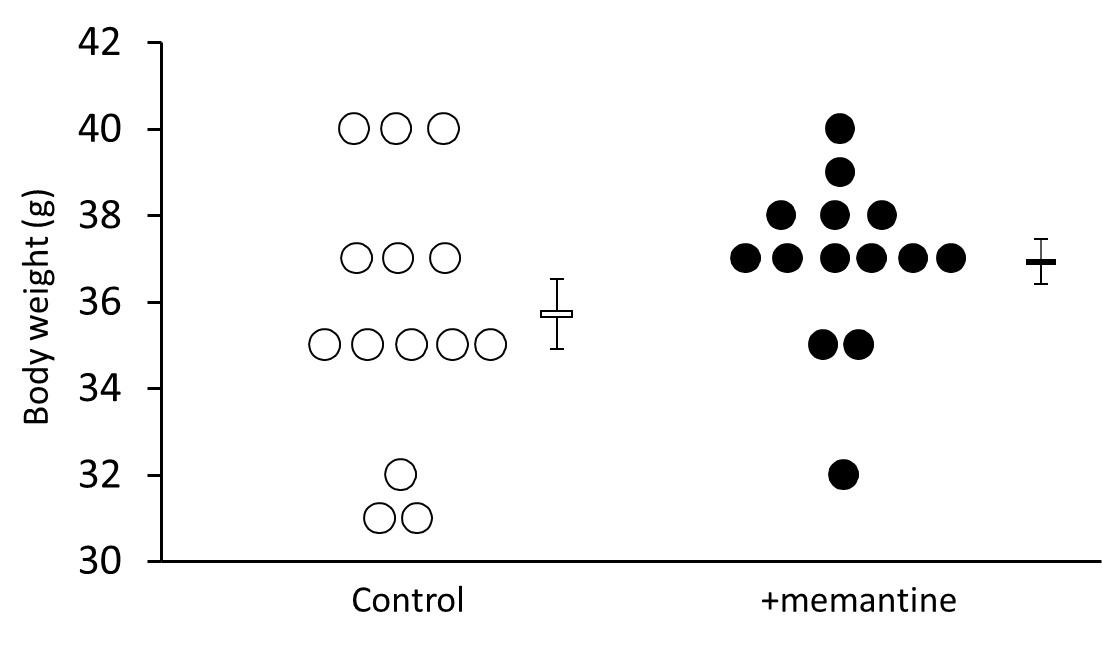


Figure S1. Title. Memantine consumption did not change the body weight of CD1 mice. Individual and averaged data points are shown as opened circles for control mice and closed circles for memantine treated mice (p > 0.05).
